# A Comparative Study of the Aggregate Index of Systemic Inflammation (AISI) and C-Reactive Protein (CRP) in Predicting Odontogenic Abscesses Severity: A Novel Approach to Assessing Immunoinflammatory Response

**DOI:** 10.3390/diagnostics14192163

**Published:** 2024-09-28

**Authors:** Marko Tarle, Marina Raguž, Ivica Lukšić

**Affiliations:** 1 Department of Maxillofacial Surgery, Dubrava University Hospital, 10000 Zagreb, Croatia; tarlemarko1@gmail.com; 2 School of Dental Medicine, University of Zagreb, 10000 Zagreb, Croatia; 3 Department of Neurosurgery, Dubrava University Hospital, 10000 Zagreb, Croatia; marinaraguz@gmail.com; 4 School of Medicine, Catholic University of Croatia, 10000 Zagreb, Croatia; 5 School of Medicine, University of Zagreb, 10000 Zagreb, Croatia

**Keywords:** odontogenic infection, systemic inflammatory indices, CRP, AISI, predictive markers, SII, LMR, PLR, NLR

## Abstract

**Background/Objectives:** Odontogenic abscesses are a common cause of emergency visits to oral and maxillofacial surgery departments and can lead to life-threatening complications if they are not recognized and treated promptly. The aim of this study was to evaluate the prognostic value of the Aggregate Index of Systemic Inflammation (AISI) in comparison to other systemic inflammatory indices, including the Systemic Immune Inflammation Index (SII), the Neutrophil-to-Lymphocyte Ratio (NLR), the Platelet-to-Lymphocyte Ratio (PLR), and the Lymphocyte-to-Monocyte Ratio (LMR), in predicting the severity of odontogenic abscesses. **Methods:** This retrospective study included 221 patients hospitalized for odontogenic abscesses at Dubrava University Hospital between January 2019 and December 2023. Clinical and laboratory data, including AISI, SII, NLR, PLR, and LMR, were collected. The severity of the abscesses was assessed using the Symptom Severity (SS) Score and patients were categorized into less severe and severe groups based on their scores. An ROC curve analysis was used to assess the predictive accuracy of each inflammatory index. **Results:** The AISI was identified as the most effective predictor of abscess severity and had the highest sensitivity (SE = 82.93) and specificity (SP = 81.63) among the indices analyzed. It outperformed C-reactive protein (CRP) in predicting severe abscesses with an AUC of 0.90 compared to 0.74 for CRP. In addition, AISI showed significant correlations with length of hospital stay and the occurrence of systemic inflammatory response syndrome (SIRS). **Conclusions:** The AISI index is a better predictor of odontogenic abscess severity compared to other systemic inflammatory markers and CRP. Its integration into clinical practice could improve the early detection of high-risk patients, leading to better treatment outcomes and lower risks of complications.

## 1. Introduction

Odontogenic infections are the most common polymicrobial infections in the oral and maxillofacial region and the main reason for the utilization of the maxillofacial emergency department [1,2,3]. The incidence of odontogenic abscesses varies depending on the population group but is particularly common in people with poor dental hygiene. According to some studies, the incidence of odontogenic abscesses in the general population is 8–10%, while it can be even higher in certain at-risk groups [4,5]. These infections usually occur as a result of untreated caries (70%), periodontal disease (gingivitis and periodontitis), or dental trauma caused by bacteria in the oral cavity. The infection usually spreads via the dental pulp to the area around the tooth root and via the alveolar ridge, “through the spongiosa and the cortical bone of the jaw into the surrounding fascial spaces”, according to the “principle of least resistance”; it initially manifests itself as cellulitis in the form of pain, swelling, and redness. If left untreated, the cellulitis develops into an odontogenic abscess. Elimination of the cause, such as tooth extraction, endodontic or periodontal therapy, and incision and drainage of the abscess, is essential for appropriate treatment [6,7]. The severity of the infection is determined by a combination of factors such as the virulence of the pathogenic bacteria, the patient’s general health and immune response, and the location and extent of the affected anatomical areas. These factors together influence the course and outcome of the infection and require an individualized approach to diagnosis and treatment [2,3,6].

The overall mortality and morbidity caused by odontogenic infections have decreased significantly over the last 50 years. However, despite advances in surgical treatment techniques and the development of antibiotics, odontogenic abscesses can lead to serious, life-threatening complications if they are not recognized and treated in time. These complications, which have a mortality rate of 10–40%, include airway obstruction, descending necrotizing mediastinitis, orbital cellulitis and abscess, septic cavernous thrombosis, cerebrospinal abscess, sepsis, systemic inflammatory response syndrome (SIRS), necrotizing fasciitis, and Lemierre’s syndrome [4,7].

In order to clinically recognize patients with more severe odontogenic abscesses that can lead to life-threatening complications and death, it is necessary to develop accurate clinical scores that can aid in the timely identification of high-risk cases. Such scores allow for a more aggressive treatment approach, including more intensive surgery, appropriate antibiotic therapy, and increased surveillance [8]. In addition to clinical symptoms and signs, laboratory parameters such as C-reactive protein (CRP), white blood cell (WBC) count, neutrophil count, lymphocyte count, and monocyte count are often used to assess the severity of an abscess [9]. However, these parameters alone are not precise enough to accurately determine the severity of the infection. This led to the development of new systemic inflammatory indices derived from the complete blood count (CBC) to assess the intensity of the immune–inflammatory response in odontogenic infections [10,11].

One of these indices is the SII (Systemic Inflammatory Index), which combines platelets, neutrophils, and lymphocytes and has been shown to be a good predictor for the development of systemic inflammatory response syndrome (SIRS) and sepsis in patients with odontogenic abscess. In addition to the SII, other scores such as NLR (Neutrophil-to-Lymphocyte Ratio), PLR (Platelet-to-Lymphocyte Ratio), AISI (Aggregate Index of Systemic Inflammation), LMR (Lymphocyte-to-Monocyte Ratio), and a combination of NLR+PLR have recently been investigated in the literature [12,13,14,15]. In particular, AISI includes neutrophils, lymphocytes, monocytes, and platelets and has been shown in the literature to be a reliable predictor of disease severity and mortality in various inflammatory diseases, including sepsis and oncologic diseases [16].

This is the first study in the literature with such a large sample of subjects to analyze the prognostic potential of these scores in assessing the severity of odontogenic abscesses on admission of patients to the hospital. The results of this study could significantly improve clinical decision making and the selection of therapeutic options, thus improving treatment outcomes in these patients.

## 2. Materials and Methods

This retrospective study was conducted on a sample of patients hospitalized for odontogenic abscesses in the period from January 2019 to December 2023 at the Dubrava University Hospital, Department of Oral and Maxillofacial Surgery, which is the largest center for oral and maxillofacial surgery in Croatia and is specialized in the surgical treatment of the most complex and severe odontogenic infections. The study was approved by the Ethics Committee of the Dubrava University Hospital (2022/1807-04, 28 July 2022), and all patients involved in the research signed a consent form.

To be included in this study, patients had to meet the following criteria: (a) over 18 years of age; (b) a clinical diagnosis of odontogenic abscess according to the ICD-10 classification that required surgical intervention [17]; (c) complete medical data from medical history in the hospital information system. The exclusion criteria were as follows: (a) presence of non-odontogenic infections; (b) presence of immune disorders; (c) receiving therapy that could significantly affect inflammatory parameters (corticosteroids, immunosuppressants, biologic therapy, non-steroidal anti-inflammatory drugs, antibiotics); and (d) incomplete medical data.

All clinical data of the patients were obtained from their medical records and the hospital’s information system. All patients were admitted to the hospital for treatment of an odontogenic abscess, and the laboratory parameters required for the calculation of systemic immune scores were obtained from the laboratory findings at hospital admission. Using data from the patients’ medical histories (clinical examinations, laboratory findings, radiologic findings) and triage examinations in the emergency department, the Symptom Severity Score (SS) was calculated according to the methodology of Sainuddin and colleagues [18]. This comprehensive score is based on several features, including clinical manifestations of systemic inflammation known as Systemic Inflammatory Response Syndrome (SIRS), laboratory parameters, and clinical parameters that are readily available to all clinicians (location of abscess, presence, and intensity of trismus and dysphagia, and signs of dehydration and presence of comorbidities) [18,19]. Based on the results of the SS score (0–18 points), patients were divided into those with a less-severe abscess (SS score of 0 to 8 points) and those with a severe abscess (SS score of 9 to 20 points) (Table 1).

Most patients were treated surgically, by extraoral and intraoral incision and drainage with insertion of a drain, which was removed after the absence of inflammatory secretion, resolution of clinical symptoms and signs of inflammation, and a decrease in inflammatory parameters, including leukocytes and CRP. During hospitalization, patients had the causative tooth removed and treatment usually included dual antibiotic therapy, usually consisting of metronidazole and amoxicillin with clavulanic acid or clindamycin in case of penicillin allergy. Antibiotic therapy was adjusted as needed based on the results of the microbiological analysis of the swab. During the surgical procedure, a swab was taken from all patients for microbiological analysis. Patients with necrotizing fasciitis, mediastinitis, and airway obstruction underwent tracheostomy, aggressive surgical and pharmacological treatment, and spent part of their hospital stay in the intensive care unit.

The systemic inflammation indices were calculated according to the following formulas: Aggregate Index of Systemic Inflammation (AISI) was calculated according to the following formula: AISI = (number of neutrophils × number of monocytes × number of platelets)/number of lymphocytes [16]. SII (Systemic Immune–Inflammation Index) was calculated according to the following formula: SII = (number of neutrophils × number of platelets)/number of lymphocytes [12]. NLR (Neutrophil-to-Lymphocyte Ratio) was calculated by dividing the number of neutrophils by the number of lymphocytes (NLR = number of neutrophils/number of lymphocytes) [13]. PLR (Platelet-to-Lymphocyte Ratio) was determined by the ratio of the number of platelets to the number of lymphocytes (PLR = number of platelets/number of lymphocytes) [14]. Finally, the LMR (Lymphocyte-to-Monocyte Ratio) was calculated as the ratio between the number of monocytes and the number of lymphocytes (LMR = number of monocytes/number of lymphocytes) [15]. These indices provide a comprehensive assessment of the inflammatory response and can be crucial in identifying patients at high risk of complications during the treatment of odontogenic abscesses (Table 2).

Statistical data processing was performed with the statistical computer program MedCalc, version 12.5.0 (MedCalc Software, Ostend, Belgium; https://www.medcalc.org accessed on 15 July 2024), and the results are presented in tables and graphs. The values of continuous variables are presented as mean ± standard deviation. The analysis of the distribution of the measured variables (Kolmogorov–Smirnov test) determined the difference in each variable’s distribution; the distribution’s normality varied from parameter to parameter. Associations (correlations) between individual parameters were examined using the Pearson test or Spearman test, depending on the normality of the data distribution. Furthermore, we have analyzed the adjusted odds ratio (aOR) using logistic regression and the hazard ratio (HR) using Cox proportional hazards regression. The potential prognostic value of the analyzed parameters was determined using a ROC (receiver operating characteristic) analysis. The test results were considered significant when *p* ≤ 0.05.

## 3. Results

In this study, a total of 221 consecutive adult patients (127 male patients, 57.5%, and 94 female patients, 42.5%) were enrolled in the study. The average age of the included patients was 41.18 ± 17.55 years, 41.71 ± 16.91 years in male patients, and 40.42 ± 18.46 years in female patients.

In the analyzed group of patients, a statistically significant association was observed between all analyzed scores and the level of seriousness of abscess measured by SS score, with especially strong associations with AISI (ρ = 0.685, *p* < 0.0001) and SIRS score (ρ = 0.719, *p* < 0.0001). There was no statistically significant association between analyzed scores and the time passed from initial dental intervention to hospitalization, as well as the time passed from symptoms occurrence to hospitalization (Table 3).

Regarding other analyzed parameters, a statistically significant association was observed between hospitalization length in days and AISI score (ρ = 0.226, *p* = 0.0007), as well as CRP levels (ρ = 0.40, *p* < 0.0001), age (ρ = 0.26, *p* = 0.0001), comorbidities (ρ = 0.205, *p* = 0.002), collection localization (ρ = 0.376, *p* < 0.0001), and complications (ρ = 0.38, *p* < 0.0001).

The most common abscess localization was perimandibular in 65.6% of cases, followed by pterygomandibular in 16.9%, and buccal in 4.5%. A statistically significant association was observed between abscess localization and patient age (ρ = 0.211, *p* = 0.001), the occurrence of dysphagia (ρ = 0.376, *p* < 0.0001), and trismus (ρ = 0.282, *p* < 0.0001), complications (ρ = 0.383, *p* < 0.0001), AISI score (ρ = 0.258, *p* = 0.0001), and CRP levels (ρ = 0.365, *p* < 0.0001).

**Figure 1:**
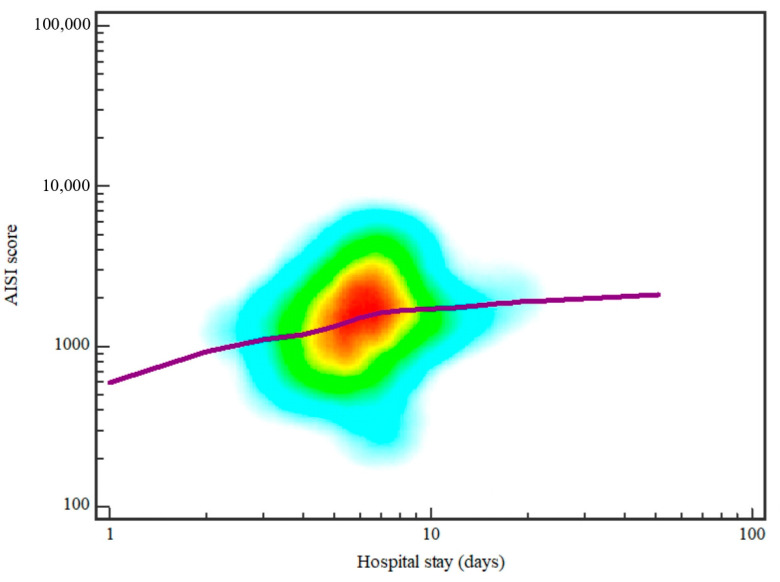
Spearman correlation between hospital stay in days and AISI score ρ = 0.226, *p* = 0.0007). The image shows a graph depicting the relationship between hospital stay (in days) on the x-axis and the AISI score on the y-axis, both on logarithmic scales. A colored density plot highlights data concentration, with red indicating the highest density. The trend line suggests a gradual increase in AISI score as the hospital stay lengthens.

Both AISI score (ρ = 0.417, *p* < 0.0001) and CRP levels (ρ = 0.357, *p* < 0.0001) showed a significant association with the occurrence of dysphagia, while only CRP showed a significant association with the trismus occurrence (ρ = 0.372, *p* < 0.0001). In addition, causative tooth and hospitalization length in days showed a significant association with the trismus occurrence (ρ = 0.297, *p* < 0.0001; ρ = 0.219, *p* = 0.001). Patient age showed a significant association with comorbidities (ρ = 0.355, *p* < 0.0001) and complication occurrence (ρ = 0.219, *p* = 0.001) (Figure 1).

There was no statistically significant association between analyzed clinical parameters and the time passed from initial dental intervention to hospitalization, as well as the time passed from symptoms occurrence to hospitalization. 

**Figure 2:**
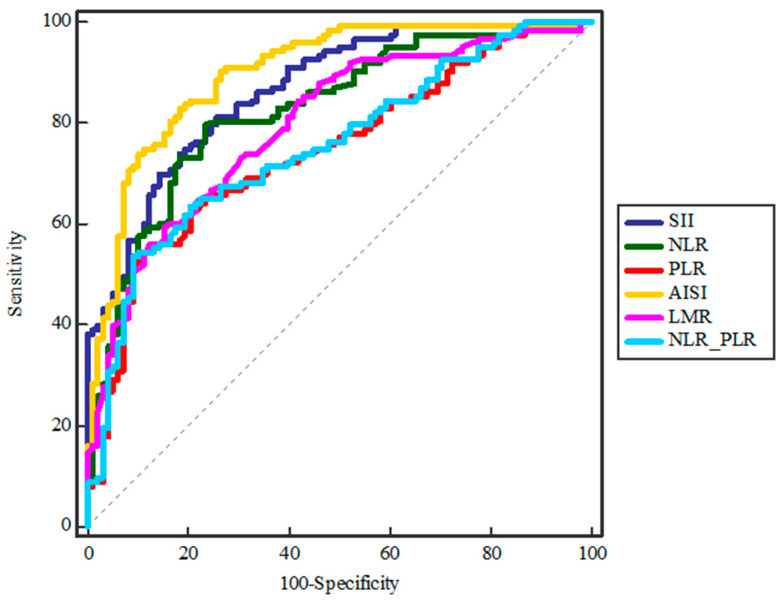
ROC analysis of inflammatory markers in predicting the severity of odontogenic abscesses. This figure shows the ROC curves for different inflammatory markers (SII, NLR, PLR, AISI, LMR, and NLR–PLR) in predicting the severity of odontogenic abscesses. The Aggregate Index of Systemic Inflammation (AISI) shows the highest predictive accuracy with an AUC of 0.90, indicating a higher sensitivity and specificity compared to other markers. The AISI curve (yellow) proves to be the most effective prognostic tool for identifying patients at high risk for severe abscesses.

Furthermore, to determine the value of the analyzed scores as possible clinical predictors of the seriousness of abscess, an ROC analysis was performed. The AISI score was shown to be a very strong indicator of abscess seriousness (SE = 82.93, SP = 81.63, AUC = 0.90, Y = 0.65, *p* = 0.0001), while the other analyzed scores were moderate to strong indicators, namely the SII score (SE = 81.30, SP = 74.49, AUC = 0.86, Y = 0.56, *p* = 0.0001), the NLR score (SE = 79.67, SP = 76.53, AUC = 0.83, Y = 0.56, *p* = 0.0001), the LMR score (SE = 59.35, SP = 84.69, AUC = 0.80, Y = 0.44, *p* = 0.0001), the PLR score (SE = 54.47, SP = 88.78, AUC = 0.75, Y = 0.43, *p* = 0.0001) and the NLR_PLR score (SE = 53.66, SP = 90.82, AUC = 0.75, Y = 0.44, *p* = 0.0001) (Figure 2).

**Figure 3:**
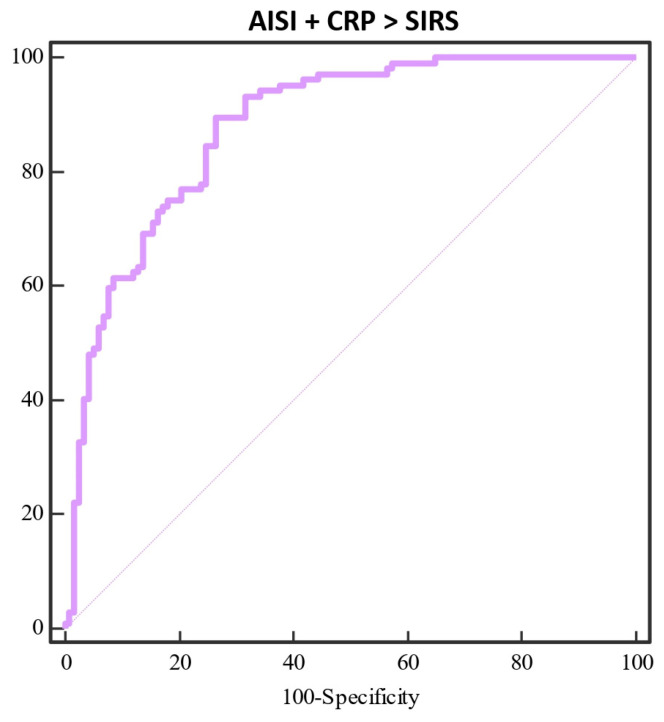
ROC curves comparing AISI and CRP in the prediction of systemic inflammatory response syndrome (SIRS). This figure presents a ROC curve for the combined systemic inflammation index (AISI) and C-reactive protein (CRP) against the prediction of systemic inflammatory response syndrome (SIRS). The combined AISI + CRP model demonstrates superior predictive accuracy with an area under the curve (AUC) of 0.88. The model exhibits a sensitivity of 89.42% and specificity of 73.50%, with a Youden index of 0.63. The statistical significance is high, with *p* < 0.0001, indicating a strong predictive performance for detecting SIRS in patients.

Additionally, the AISI score (SE = 85.58, SP = 77.78, AUC = 0.88, Y = 0.64, *p* < 0.0001) and CRP level (SE = 73.08, SP = 67.52, AUC = 0.74, Y = 0.41, *p* < 0.0001) were analyzed to SIRS, both having significant value; still, the AISI score showed to have stronger prognostic value. Together, the AISI and CRP values showed to be significant prognostic factors (SE = 89.42, SP = 73.50, AUC = 0.88, Y = 0.63, *p* < 0.0001) (Figure 3).

**Figure 4:**
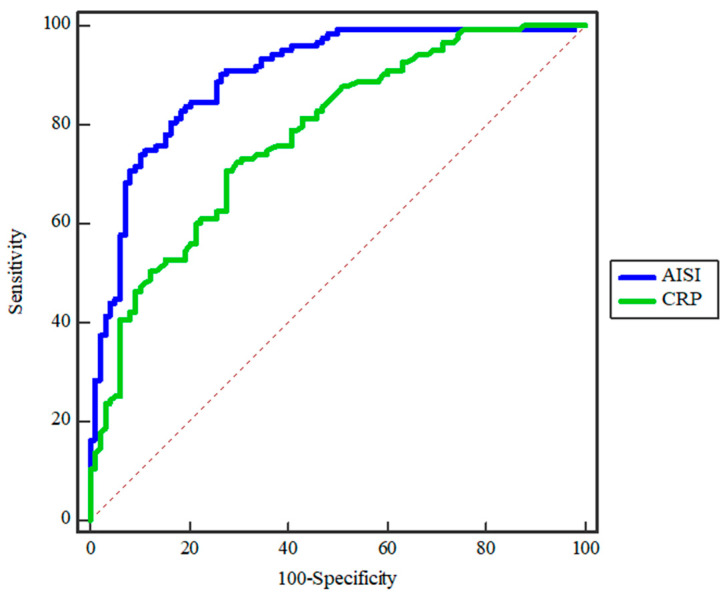
ROC curve analysis comparing AISI and CRP in predicting abscess severity. This figure presents a comparison of the ROC curves for the Aggregate Index of Systemic Inflammation (AISI) and C-reactive protein (CRP) in predicting the severity of odontogenic abscesses. The AISI (blue curve) shows a significantly higher predictive accuracy with an area under the curve (AUC) of 0.90, sensitivity of 82.93%, specificity of 81.63%, Youden index (Y) of 0.65, and a *p*-value of 0.0001. In contrast, CRP (green curve) has an AUC of 0.78, with a Youden index of 0.43 and a *p*-value of 0.0001, indicating that AISI is a superior predictor of abscess severity compared to CRP, offering better clinical utility in assessing and managing high-risk patients.

When comparing the analysis of the AISI score (SE = 82.93, SP = 81.63, AUC = 0.90, Y = 0.65, *p* = 0.0001) and CRP levels (SE = 70.73, SP = 72.45, AUC = 0.78, Y = 0.43, *p* = 0.0001), it is clear that AISI score has significant prognostic value in predicting the seriousness of an abscess (Figure 4). Moreover, the AISI and CRP values together showed to be significant prognostic factors (SE = 78.23, SP = 76.46, AUC = 0.91, Y = 0.51, *p* < 0.0001) (Figure 4).

Moreover, we have calculated the cut-off value for the AISI score to be 1522.2. This means that patients with an AISI score above 1522.2 are more likely to have a severe condition, while those with lower scores are at a lower risk.

In addition, we analyzed the HR and aOR with 95% confidence intervals (CIs) for the SII, AISI, LMR, NLR, PLR, and NLR_PLR score, SS score, age, and sex. 

SII score showed a slight but statistically significant increase in hazard (HR = 1.0003, 95% CI = 1.0000 to 1.0005), while LMR score significantly reduces hazard, indicating a protective effect (HR = 0.6278, 95% CI = 0.4674 to 0.8434). The findings for the AISI score (HR = 1.000, 95% CI = 0.9999 to 1.0001) and patient age (HR = 0.989, 95% CI = 0.9772 to 1.0003) suggest that neither of them are a significant predictor of the event’s risk. HR estimates for NLR, PLR, and NLR_PLR score are highly unstable and unreliable, with no statistically significant impact on hazard. The HR for sex (HR = 1.153, 95% CI = 0.7936 to 1.6748) suggests that male patients have a 15.29% higher risk of the event occurring than female patients. However, this finding is not statistically significant. The HR for SS score (HR = 1.2864, 95% CI = 1.1979 to 1.3813) indicates that, for each unit increase in the SS score, the risk of the event increases by 28.64%, and this result is statistically significant. This suggests that the SS score strongly predicts the event risk. 

Regarding aOR analysis, AISI shows a slight but statistically significant increase in the odds of the event. For each unit increase in AISI, the odds of the event increase by 0.23% (aOR = 1.0023, 95% CI = 1.0016 to 1.0030), while the SII score has a negligible impact on the odds of the event, and the result is not statistically significant, indicating it may not be a strong predictor (aOR = 1.0011, 95% CI = 0.9998 to 1.0023). This suggests that AISI is a reliable predictor of disease severity. A higher LMR score was associated with a 65.38% reduction in the odds of the event occurring (aOR = 0.3462, 95% CI = 0.2442 to 0.4907); the result is statistically significant, indicating that LMR is a protective factor. NLR (aOR = 94.5 × 10^24^, 95% CI = 8.8753 × 10^−9^ to 1.0054 × 10^6^) and PLR score (aOR = 94.3 × 10^24^, 95% CI = 8.9721 × 10^−9^ to 991.3018 × 10^57^) showed an extremely high odds ratio, while NLR_PLR score (aOR = 1.06 × 10^−26^, 95% CI = 1.0063 × 10^−60^ to 111,319,854.6607) showed an extremely low odds ratio with a wide confidence interval, providing an indication these estimates are unreliable. For each additional year of age (aOR = 0.9901, 95% CI = 0.9694 to 1.0113), the odds of the event decrease by approximately 0.99%, but this reduction is not statistically significant. Although sex (aOR = 1.5855, 95% CI = 0.7554 to 3.3279) indicates that male patients have 58.55% higher odds of the event occurring than female patients, the results are not statistically significant.

## 4. Discussion

Odontogenic abscesses are one of the most common reasons why patients come to the maxillofacial emergency department and thus the most common infection in the oral and maxillofacial region. Although the incidence of odontogenic infections is decreasing significantly and is being successfully treated, the worrying fact is that complications of odontogenic abscesses, such as fascial space infections, can be serious and life-threatening due to the connection of the fascial spaces of the neck with vital structures [1,2,3]. These complications are usually caused by delays in treatment, lack of recognition, and inadequate treatment, especially in people with comorbidities such as diabetes, immunocompromised conditions, obesity, or chronic diseases. Mortality due to complications of odontogenic infections varies but can range from 10 to 40% if they are not recognized and treated in time. It is therefore crucial to recognize the severity of the infection in time and to predict possible complications [4,8,20].

The decision to treat odontogenic abscesses is based on clinical assessment, radiographic findings, and laboratory parameters. However, it is sometimes difficult for clinicians to assess the severity of the infection, as clinical signs may appear later or be less pronounced, which may lead to an incorrect clinical assessment and inappropriate therapy, either surgical or pharmacological [9]. For this reason, it is necessary to discover and develop additional predictors for the severity and course of the disease in order to be able to react in a timely manner in the treatment process.

C-reactive protein (CRP) is the most commonly used inflammatory marker in the assessment of disease severity, including odontogenic infections, as it responds quickly to inflammation and is able to rapidly detect changes in the patient’s condition. CRP has a very short half-life of 5–7 h, unlike leukocytes (5–6 days), and peaks after 24 h, making it a very sensitive marker of inflammation and its progression. Due to its short half-life, changes in CRP can quickly reflect the response to treatment, allowing the effectiveness of therapy to be monitored [9,11,21].

In recent years, however, numerous inflammatory indices have been developed, such as SII (Systemic Inflammatory Index), NLR (Neutrophil-to-Lymphocyte Ratio), PLR (Platelet-to-Lymphocyte Ratio), LMR (Lymphocyte-to-Monocyte Ratio), and combined NLR–PLR, which allow a more complex and robust assessment of the immune response. These indices include multiple components of the immune response, such as neutrophils, lymphocytes, and platelets, and are often associated with better prediction of patient outcome and prognosis. These parameters provide a comprehensive overview of various aspects of the body’s immune response to inflammation, which can allow for a more accurate prediction of disease severity [12,13,14,15].

Although CRP is specific to the acute phase, it does not take other components that can provide additional information about the patient’s condition into account. Studies that have examined the prognostic significance of the above inflammatory indices have relied on single indices [10,11,22,23]. In our study, which involved 221 patients, we analyzed different systemic inflammatory indices (AISI, SII, NLR, PLR, LMR, NLR–PLR) and their influence on the severity of odontogenic abscesses. This is the first study in the literature to investigate the prognostic significance of all the mentioned immunologic scores on the severity of odontogenic abscesses, in contrast to previous studies that focused on one or two inflammatory indices [10,11,22,23]. Our study is more informative because it analyzes all indices in the same group of patients and identifies those that are the best predictors. This comprehensive approach allows for more accurate prediction of outcomes and provides a deeper understanding of the immune responses associated with odontogenic infections. In addition, this is the first study to include the Aggregate Index of Systemic Inflammation (AISI) in the analysis. This is a first in the study of odontogenic infections, as this index has not been studied in this context before.

The hematologic cell index SII, which is based on neutrophil, platelet, and lymphocyte counts, has been shown in numerous studies to be a very valuable biomarker for predicting poor outcomes in cancer, cardiovascular disease, autoimmune disease, liver disease, and in patients with COVID-19 [12,24,25,26,27,28]. Several studies have analyzed the systemic inflammatory indices in patients with odontogenic infections. Roi et al. conducted a retrospective study of 39 patients with odontogenic cervicofacial phlegmons in which they examined the changes in SII, NLR, CRP, and WBC before and after surgical and pharmacological treatment. The results showed a significant reduction in these parameters after treatment, indicating their potential value for assessing the severity of inflammation and monitoring treatment success [10]. Another study by Pricop et al. investigated the prognostic value of SII and Symptom Severity Score (SS) in 108 patients hospitalized for odontogenic infections. Their results showed that SII and SS were reliable predictors of the development of sepsis and systemic inflammatory response syndrome (SIRS) in these patients, with the accuracy of SII being particularly high in predicting sepsis [23].

Over the last 10 years, the NLR index has been the subject of many biomedical studies. It consists of the ratio of neutrophils, representatives of non-specific immunity, and lymphocytes, representatives of specific immunity. The NLR is a well-known prognostic indicator that correlates independently with mortality both in the general population (HR 1.14, 95% CI 1.10–1.17, per quartile of NLR) and in certain disease groups such as sepsis, pneumonia, COVID-19, cancer, and others [29]. Numerous studies have investigated the prognostic value of NLR in odontogenic infections. In 2024, Ghasemi et al. concluded in their systematic review of a sample of nine studies that an elevated Neutrophil-to-Lymphocyte Ratio (NLR) correlates significantly with the severity of odontogenic infections, length of hospital stay and risk of complications [30]. In their study on a sample of 108 patients, Rosca et al. concluded that the combination of C-reactive protein (CRP) and NLR is a reliable and accessible biomarker for predicting the severity of odontogenic infections compared to their individual prediction [11].

PLR (Platelet-to-Lymphocyte Ratio) is used as a prognostic marker in acute bacterial and odontogenic infections. Platelets play a key role in the inflammatory response in bacterial infections through the activation and release of anti-inflammatory mediators, interaction with leukocytes, and the formation of platelet-leukocyte aggregates, which contributes to a rapid immune response to the infection. An increased PLR reflects this increased platelet inflammatory activity, along with a decrease in lymphocyte count, which is associated with a more severe form of the disease. In odontogenic infections, PLR has been shown to be useful in assessing the severity of disease and can help in decisions about urgent therapeutic intervention, particularly in cases with sepsis or deep abscesses [14,31]. In their study of a sample of 271 patients, Kusumoto et al. concluded that the Platelet-to-Lymphocyte Ratio (PLR), along with other hematologic and inflammatory parameters, may be useful as an additional diagnostic tool for the early detection of severe odontogenic infections, including necrotizing soft tissue infections and deep neck abscesses [32].

LMR (monocyte-to-lymphocyte ratio) is an inflammatory marker that reflects the balance between the inflammatory response (monocytes) and adaptive immunity (lymphocytes) [33]. In odontogenic infections, a lower LMR may indicate a more severe form of the disease with a stronger inflammatory response, which may predict a higher risk of complications such as sepsis. Our study showed a statistically significant negative correlation of LMR with the severity of odontogenic abscess and a moderate to strong predictive property for reduced LMR values (SE = 59.35, SP = 84.69, AUC = 0.80, Y = 0.44, *p* = 0.0001). This suggests that monocytes play an important role in acute polymicrobial bacterial inflammation. To our knowledge, this is the first study to investigate the influence of the LMR index in odontogenic infections.

Our research is valuable because it examines the potential utility of the above inflammatory indices in a large sample of subjects and provides insight into which of them may be most useful in assessing the severity of odontogenic abscesses and the incidence of fatal complications. While previous studies have primarily focused on single indices, such as CRP in assessing disease severity, our approach is novel as it examines multiple indices within the same cohort [11,30]. This allows for a comprehensive assessment of their predictive capabilities. For instance, prior research has highlighted the significance of the SII in predicting adverse outcomes in patients with infections [23] and the NLR as a reliable prognostic indicator [30]. By analyzing these indices alongside AISI, we provide a more robust understanding of the inflammatory response in patients with odontogenic abscesses, which has not been thoroughly explored in the existing literature. Although prior research has established CRP as a key inflammatory marker in assessing the severity of infections, including odontogenic abscesses [11,30], our findings suggest that the AISI provides superior predictive value for abscess severity. By integrating AISI with other indices, like the SII and the NLR [23,30], we can offer a more robust analysis of the immune response in patients with odontogenic abscesses. Furthermore, when comparing our results with previous papers [10,23], our findings align with or differ from those of existing research and contribute to a broader understanding of inflammatory markers in this context. By integrating insights from these studies, we aim to provide a more comprehensive perspective on the predictive value of various inflammatory indices, including the AISI, in assessing the severity of odontogenic abscesses.

In light of the findings of previous studies, we wanted to see how the AISI score, which additionally includes monocytes that play an important role in acute bacterial inflammation, could predict outcome. Unlike other hematologic inflammation indices, the AISI uses four types of blood cells involved in the inflammatory process. It was first described in 2018 by Paliogiannis and colleagues, who investigated it as a preoperative predictor of length of hospital stay in open elective thoracic surgery and showed that it is a good predictor of the risk of prolonged hospitalization [34]. In addition, previous studies have shown that AISI is an important prognostic marker not only in cardiovascular disease and COVID-19, but also in the context of lung diseases, including chronic obstructive pulmonary disease (COPD) and idiopathic pulmonary fibrosis (IPF) [16,35,36]. In patients with IPF, the AISI has been shown to be useful in assessing disease severity and mortality risk, suggesting its value in monitoring systemic inflammation and associated clinical outcomes [16]. In our study, the AISI was the best predictor of abscess severity, with the highest sensitivity (SE = 82.93) and specificity (SP = 81.63) among the indices analyzed, while the SII had a similar but slightly lower predictive value. The AISI outperformed the SII as a predictor of abscess severity, which could be explained by the fact that the AISI includes all key components of the acute inflammatory response in its formula.

Neutrophils are the body’s first line of defense against invading pathogens. This occurs through mechanisms such as chemotaxis, phagocytosis, release of reactive oxygen species (ROS) and granular proteins, and the synthesis and release of cytokines. In addition to this role, neutrophils are the major effector elements in the systemic inflammatory response (SIRS) and additionally recruit, activate, and program other immune cells through the secretion of proinflammatory and immunomodulatory cytokines and chemokines. After monocytes have migrated to the site of infection, they differentiate into macrophages and dendritic cells, which are essential for phagocytosis of pathogens and the elimination of damaged cells. They also release anti-inflammatory cytokines such as TNF-α, IL-1β, and IL-6, which promote the recruitment of further immune cells and amplify the inflammatory response [37]. Lymphocytes, on the other hand, are responsible for a specific immune response. Their lower levels in the AISI formula may indicate suppressed adaptive immune function, which can lead to a more severe infection. The above processes point to the complexity of acute odontogenic inflammation and the importance of the AISI score, which is the most complex inflammation score to date. As CRP has so far proven to be a very sensitive inflammatory marker, it best predicts the severity of odontogenic abscesses in combination with the AISI score. In addition, the AISI proved to be a good predictor of SIRS. This detailed integration makes the AISI more sensitive to changes in the patient’s immune status, especially in severe infections such as odontogenic abscesses.

Although all mentioned hematologic biomarkers together with clinical and radiologic findings can help to assess the severity of odontogenic infections and thus prevent the occurrence of fatal complications, the AISI proved to be a very good potential inflammatory marker in this study. 

By improving the predictive accuracy of severity assessment, we aim to facilitate better clinical decision making and enhance patient outcomes. The incorporation of AISI into routine evaluations could allow clinicians to identify high-risk patients earlier, leading to timely interventions that may prevent complications. Furthermore, this approach may help to standardize the assessment of inflammatory responses in odontogenic infections, ultimately contributing to more personalized and effective treatment strategies. By elucidating the relationship between inflammatory indices and clinical severity, our findings could also inform future research directions and guidelines for managing odontogenic abscesses. However, further research is needed to understand its full potential in this area.

This study has several limitations that should be considered when interpreting the findings. The retrospective nature of the study relies on existing medical records, which may introduce a lack of controlled data collection and selection bias. Conducting the study at a single institution may limit the generalizability of the results to other populations or clinical settings. Although the study includes 221 patients, a larger sample size may be necessary to validate the findings and enhance the robustness of the statistical analysis. Other variables influencing the severity of odontogenic abscesses, such as patient comorbidities, socioeconomic factors, and variations in treatment protocols, might not have been fully accounted for. The findings require validation in external cohorts to confirm the predictive utility of the AISI across diverse clinical contexts. Acknowledging these limitations is crucial for understanding the implications of our findings and their potential impact on clinical practice.

## 5. Conclusions

Our results suggest that the new, easy-to-measure AISI score is an extremely useful tool for the clinical assessment of abscess severity, outperforming not only the SII but also other non-inflammatory scores and CRP. This index can help identify patients who require more intensive treatment, including more aggressive surgery, antibiotic therapy, and extraction of the causative tooth, thereby improving treatment outcomes and reducing the risk of complications.

## Figures and Tables

**Table 1:** The Symptom Severity Score (SS) for odontogenic infections, as developed by Sainuddin et al. [18].

Criteria		Score	Max Score
Systemic Inflammatory Response Syndrome (SIRS)	Temperature > 38.3 °C	1	4
	Heart rate > 90 bpm	1	
	RR 20/min	1	
	WBC < 4 or >12 × 10^9^	1	
Thrismus	Moderate < 2 cm	3	4
	Severe < 1 cm	4	
Dysphagia	Mild—able to swallow most foods	2	5
	Moderate—unable to swallow fluids	4	
	Severe—drooling saliva	5	
Collection in 1 fascial space	Low severity (canine, vestibular)	1	5
	Moderate severity (buccal)	2	
	High severity (all other spaces)	4	
Collection in 2 or more fascial spaces		5	
Sign of dehydration (BP/Urea/Skin turgor)		1	2
Comorbidities: diabetes mellitus, immunocompromised status, known or suspected chronic alcohol misuser		1	
**Total Score**			**20**

SIRS, Systemic Inflammatory Response Syndrome; BP, blood pressure; RR, respiratory rate; WBC, white blood cells.

**Table 2:** Demographic, clinical, and laboratory data of patients with odontogenic abscesses based on infection severity according to Symptom Severity Score (SS Score).

	Mild Odontogenic Abscesses (SS Score ≤ 8; *n* = 98)	Severe Odontogenic Abscesses (SS Score ≥ 9; *n* = 123)	*p*-Value
Age, years	41.5 (18–80)	33 (18–85)	0.10
Sex, male/female	52/46	75/48	0.95
Hospital stay, days	5.6 ± 2.8	7.94 ± 5.6	0.0003
Previous dental intervention, yes/no	25/73	38/85	0.2900
Comorbidities, yes/no	28/70	34/89	1.0
Days from symptom onset to hospitalization	3.5 (1–16)	4 (1–21)	0.41
Leukocytes (×10^9^/L)	11.25 (6–18)	14.7 (6.9–26.4)	<0.0001
Platelets (×10^9^/L)	236.5 (112–482)	267 (143–594)	0.001
Neutrophils (×10^9^/L)	8.35 (3–16.3)	11.8 (5.3–27.2)	<0.0001
Lymphocytes (×10^9^/L)	1.65 (0.3–3.8)	1.3 (0.2–3.4)	<0.0001
Monocytes (×10^9^/L)	0.7 (0.1–1.9)	0.9 (0.1–2.5)	<0.0001
SII	1178.6 (267.3–2878.1)	2343 (501.9–17,256.9)	<0.0001
NLR	5 (1.3–20.7)	9.1 (2.3–68)	<0.0001
PLR	144.9 (62–396)	220.9 (86.8–910)	<0.0001
AISI	815.7 (106.9–4005.6)	1978.3 (200.8–12,079.8)	<0.0001
LMR	2.5 (0.83–6.67)	1.4 (0.42–6)	<0.0001
NLR + PLR	152 (63.9–414)	229.7 (93.5–978)	<0.0001
SIRS	0 (0–3)	2 (0–4)	<0.0001
SS score	6 (2–8)	11 (9–18)	<0.0001
CRP	66.8 (2.6–270.3)	146.1 (18.2–450)	<0.0001

Data are presented as mean ± standard deviation or median. SII, Systemic Inflammatory Index; NLR, Neutrophil-to-Lymphocyte Ratio; PLR, Platelet-to-Lymphocyte Ratio; AISI, Aggregate Index of Systemic Inflammation; LMR, Lymphocyte-to-Monocyte Ratio; SIRS, Systemic Inflammatory Response Syndrome; SS score, Symptom Severity Score; CRP, C-reactive protein.

**Table 3:** Association between inflammatory indices with clinical parameters. The table illustrates the correlations between various inflammatory indices and clinical parameters. Warm colors, ranging from yellow to red, indicate the strength of positive correlations, while cool colors, ranging from green to blue, represent negative correlations between the examined parameters. Each box displays the upper value as the strength of the correlation (ρ) and the value below it as the *p*-value, providing insight into the significance of these associations.

SII		0.879 <0.0001	0.637 <0.0001	0.827 <0.0001	0.809 <0.0001	0.628 <0.0001	0.809 <0.0001	0.666 <0.0001	0.465 <0.0001	0.018 *p* = 0.792	0.003 *p* = 0.969	−0.537 <0.0001
NLR	0.879 <0.0001		0.569 <0.0001	0.675 <0.0001	0.648 <0.0001	0.560 <0.0001	0.752 <0.0001	0.634 <0.0001	0.516 <0.0001	0.025 0.709	−0.047 0.485	−0.680 <0.0001
SS score	0.637 <0.0001	0.569 <0.0001		0.488 <0.0001	0.474 <0.0001	0.864 <0.0001	0.651 <0.0001	0.738 <0.0001	0.548 <0.0001	−0.001 0.992	0.018 0.794	−0.461 <0.0001
NLR+PLR	0.827 <0.0001	0.675 <0.0001	0.488 <0.0001		0.999 <0.0001	0.435 <0.0001	0.504 <0.0001	0.409 <0.0001	0.297 <0.0001	0.000 0.999	0.050 0.461	−0.378 <0.0001
PLR	0.809 <0.0001	0.648 <0.0001	0.474 <0.0001	0.999 <0.0001		0.422 <0.0001	0.484 <0.0001	0.390 <0.0001	0.284 <0.0001	−0.002 0.980	0.052 0.444	−0.358 <0.0001
Severity	0.628 <0.0001	0.560 <0.0001	0.864 <0.0001	0.435 <0.0001	0.422 <0.0001		0.685 <0.0001	0.719 <0.0001	0.477 <0.0001	0.030 0.659	0.017 0.804	−0.510 <0.0001
AISI	0.809 <0.0001	0.752 <0.0001	0.651 <0.0001	0.504 <0.0001	0.484 <0.0001	0.685 <0.0001		0.718 <0.0001	0.497 <0.0001	0.016 0.814	−0.020 0.773	−0.806 <0.0001
SIRS	0.666 <0.0001	0.634 <0.0001	0.738 <0.0001	0.409 <0.0001	0.390 <0.0001	0.719 <0.0001	0.718 <0.0001		0.440 <0.0001	−0.022 0.749	−0.006 0.929	−0.520 <0.0001
CRP	0.465 <0.0001	0.516 <0.0001	0.548 <0.0001	0.297 <0.0001	0.284 <0.0001	0.477 <0.0001	0.497 <0.0001	0.440 <0.0001		−0.061 *p* = 0.366	−0.085 *p* = 0.206	−0.402 <0.0001
Dental intervention	0.018 0.792	0.025 0.709	−0.001 0.992	0.000 0.999	−0.002 0.980	0.030 0.659	0.016 *p* = 0.814	−0.022 *p* = 0.749	−0.061 *p* = 0.366		−0.093 *p* = 0.169	−0.017 *p* = 0.800
Symptom onset	0.003 0.969	−0.047 *p* = 0.485	0.018 *p* = 0.794	0.050 *p* = 0.461	0.052 *p* = 0.444	0.017 *p* = 0.804	−0.020 0.773	−0.006 0.929	−0.085 0.206	−0.093 0.169		0.011 0.868
LMR	−0.537 <0.0001	−0.680 <0.0001	−0.461 <0.0001	−0.378 <0.0001	−0.358 <0.0001	−0.510 <0.0001	−0.806 <0.0001	−0.520 <0.0001	−0.402 <0.0001	−0.017 0.800	0.011 0.868	
	SII	NLR	SS score	NLR+PLR	PLR	Severity	AISI	SIRS	CRP	Dental intervention	Symptom onset	LMR

SII, Systemic Inflammatory Index; NLR, Neutrophil-to-Lymphocyte Ratio; SS score, Symptom Severity Score; PLR, Platelet-to-Lymphocyte Ratio; AISI, Aggregate Index of Systemic Inflammation; SIRS, Systemic Inflammatory Response Syndrome; CRP, C-reactive protein; LMR, Lymphocyte-to-Monocyte Ratio.

## Data Availability

All data generated or analyzed during this study are included in this published article.
